# Supplementary material for: Examining pulmonary TB patient management and healthcare workers exposures in two public tertiary care hospitals, Bangladesh
Source: PLOS Glob Public Health. 2022 Jan 5;2(1):e0000064. doi: 10.1371/journal.pgph.0000064 (PMC10021262; doi:10.1371/journal.pgph.0000064)
Supplement: Attachment — Submitted filename: Response to reviewers comments_OCT 14.docx [file pgph.0000064.s001.docx]

Date: October 14, 2021

To

Raquel Muñiz-Salazar, Ph.D.
Academic Editor
PLOS Global Public Health

Re - Examining pulmonary TB patient management and healthcare workers exposures in two public tertiary care hospitals, Bangladesh - PGPH-D-21-00420

Dear Raquel Muñiz-Salazar:

We are thankful to the reviewers for their valuable feedback. We are also grateful to the editor for allowing us to respond to the comments. Based on the helpful feedback, we revised the manuscript and believe it is more precise, clear, and informative. The following is an itemized list of our specific responses to the reviewers’ comments. We have highlighted where the changes have been made in the marked version of the manuscript.

We would appreciate your further review. Please contact me directly with any additional questions or comments. We look forward to hearing from you.

Sincerely

Md. Saiful Islam

Corresponding Author

saiful@icddrb.org

**Comments:** Thank you for including your ethics statement. “We sought informed written consent before the interviews. The institutional review board of the International Centre for Diarrheal Diseases Research, Bangladesh (icddr,b), and the University of New South Wales ethical review committee approved the study (PR-16090 and HC# HC180517).” In your ethics statement, please also state whether written informed consent was obtained from the patients whose medical records were observed or participants included in the observational sessions. If the ethics committee waived the need for informed consent in these cases, please state this.

***Response:*** *We sought informed written consent from hospital directors and unit heads to review the medical records. The behaviours and practices we observed in the hospital wards were considered public behaviours, and the ethics committee of icddr,b and UNSW waived the need for informed consent from the participants in this case.*

**Comments:** 2. Please provide separate figure files in .tif or .eps format only.

***Response:*** *All the figures have been provided in .tiff. format.*

**Comments:** Since your data is not available for proprietary reasons, please explain via email why the data is not available. Please also include the contact information for the third party organization that should be contacted should other researchers want to request access to this data and please include the full citation of where the data can be found. We also request that you verify with us via email that any researcher will be able to obtain the data set in the same manner that the you have obtained it. If you feel you are unwilling or unable to adhere to this policy, please explain your reasons by return email and your exemption request will be escalated to the editor for approval. Your exemption request will be handled independently and will not hold up the peer review process, but will need to be resolved should your manuscript be accepted for publication. One of the Editorial team will be in touch if they require more information.

***Response:*** *Data will not be freely available in public repositories due to ethical restrictions on protecting study participants privacy. One copy of the complete data set will remain at icddr,b in accordance with its policies and guidelines. The entire dataset (anonymized and decoded) and metadata will be stored at icddr,b data repository system after completion of the study. Data access will be subject to the icddr,b data policy (*[***http://www.icddrb.org/policies***](http://www.icddrb.org/policies)*) upon approval from the institutional review board. Interested parties may contact Ms Armana Ahmed (aahmed@icddrb.org) with further inquiries related to data access.*

**Comments:** Please amend your detailed Financial Disclosure statement. This is published with the article, therefore should be completed in full sentences and contain the exact wording you wish to be published.i) State the initials, alongside each funding source, of each author to receive each grant.

**Response:** *Thank you. We added following statement, “This research protocol was funded by the United States Centre for Disease Control and Prevention (CDC), through the cooperative agreement grant number 5U01GH1207. MSI and SB received this award. The funders had no role in study design, data collection and analysis, decision to publish, or preparation of the manuscript.*

**Comment:** Please review your reference list to ensure that it is complete and correct. If you have cited papers that have been retracted, please include the rationale for doing so in the manuscript text, or remove these references and replace them with relevant current references. Any changes to the reference list should be mentioned in the rebuttal letter that accompanies your revised manuscript. If you need to cite a retracted article, indicate the article’s retracted status in the References list and also include a citation and full reference for the retraction notice.

***Response:*** *The reference list has been thoroughly checked for errors. We did not cite any paper that has been retracted from publication.*

**Review Comments to the Author**

**Reviewer #1**

**Comment:** In their manuscript, titled “Examining pulmonary TB patient management and healthcare workers exposures in two public tertiary care hospitals, Bangladesh”, Islam and colleagues have provided a wide range of evidence for implementing tuberculosis (TB) infection prevention and control (IPC) in public tertiary care general hospitals in Bangladesh. Their study uses several methods, including direct observation, a detailed review of patient records, and in depth interviews with hospital staff in two study sites in order to gain an understanding of current TB management. Their results reveal that current practice is indeed insufficient to curb TB transmission, especially in inpatient wards. The study’s utility in informing IPC guidelines is clear; the authors conclude by recommending a comprehensive set of actions to minimize TB transmission, which may be generalized to other healthcare settings in Bangladesh and other resource-limited contexts. Following some methodological clarification and additional analysis, which I elaborate on below, this study will provide a clear case for TB IPC implementation in public tertiary care hospitals in Bangladesh.

***Response:*** *Thank you so much for the thorough review of the manuscript, valuable input, feedback, and appreciation.*

**Minor Comments:** There are several instances in each section of the manuscript where I suggest adjusting basic wording and punctuation in order to meet PLoS guidelines for clarity; each of these instances is listed below.

**Abstract:**

**Comment:** In the first line of the introduction, please replace “TB” with “tuberculosis (TB)”.

***Response:*** *We replaced “TB” with “tuberculosis (TB). Now it reads, “Implementation of tuberculosis (TB) infection prevention and control (IPC) guidelines in public tertiary care general hospitals remain challenging due to limited evidence of pulmonary TB (PTB) patients’ duration of hospital stay and management” on page 2, line 26.*

**Comment:** In the last line of the introduction, please add replace “hospitals, Bangladesh” with “hospitals in Bangladesh”.

***Response:*** *We revised the phrase. Now it reads, “To fill this evidence gap, this study examined adult PTB patient management, healthcare workers’ (HCWs) exposures and IPC practices in two public tertiary care hospitals in Bangladesh”* on page 2, line 30.

**Comment:** In the results section, please replace “contaminate” with “contaminated” in the first sentence.

***Response:*** *We have replaced “contaminate” with “contaminated” in the first sentence. Now it reads,* *“Over 20 months, we identified 1,200 presumptive TB patients through the hospital record review, of whom 263 were confirmed PTB patients who stayed in the hospital, a median of 4.7 days without TB treatment and possibly contaminated the inpatients wards”* on page 2, line 36.

**Comment:** In the results section, please end the sentence after the word “mask”. Then, the following sentence should be, “Further, patients did not wear any respirators”.

***Response:*** *We corrected accordingly. Now it reads, “Further, patients and HCWs did not wear any respirators”* on page 2, lines 41-42.

**Comment:** In the conclusion, consider adding “for some duration of time” to the end of the first sentence. Saying that most TB patients stayed in the hospitals untreated implies that patients always remained untreated throughout their entire stay.

***Response:*** *Corrected accordingly. Now it reads, “The study identified that most TB patients stayed in the hospitals untreated for some duration of time”* on page 2, lines 43-44.

**Introduction:**

**Comment:** In Line 6, please replace “drug-resistance” with “drug-resistant”.

***Response:*** *We have replaced “drug-resistance” with “drug-resistant”. Revised statement is, “Due to limited implementation of TB infection prevention and control (IPC) healthcare measures in some LMIC settings, healthcare facilities are now a common infection source with drug-sensitive and drug-resistant strains of TB”* on page 4, line 73.

**Comment:** In Line 10, consider removing the sentence that starts with “Diagnostic delay”, as the same thought is conveyed two sentences later.

***Response:*** *The sentence has been deleted on page 4, line 77.*

**Comment:** In Line 12, please start the sentence with “A lack of screening procedures”. In Line 14, please add an apostrophe after wards: “hospital wards’ occupants”.

***Response:*** *Thank you. The revised statement is, “A lack of screening procedures for patients with presumptive TB on admission may lead to a delayed diagnosis that allows the opportunity for nosocomial TB transmission from untreated PTB patients to other hospital wards’ occupants”* on page 4, line 79-81*.*

**Comment:** In Line 23 (top of Page 5), please replace “co-morbid TB patients” with “TB patients with co-morbidities”.

***Response:*** *The sentence has been revised. Now it reads, “In Bangladesh, tertiary care general hospitals admit presumptive TB patients for diagnosis and occasionally provide treatment to TB patients with co-morbidities” on page 5, line 90.*

**Comment:** In Lin 25, please add an apostrophe after healthcare workers so that it reads “healthcare workers’ (HCW)”.

**Response:** We have corrected this *on page 5, line 92.*

**Comment:** In Line 26, please add “Bangladesh” so that it reads “in Bangladesh’s public tertiary care”.

***Response:*** *The sentence has been revised. Now it reads, “Due to the lack of evidence about PTB patients’ stay in Bangladesh’s public tertiary care general hospitals, the implementation of TB IPC measures in these facilities remained challenging” on page 5, lines 93-95.*

**Comment:** In Line 27, please add an apostrophe after patients so that it read “TB patients’ stay”

***Response:*** *An apostrophe has been added after patients, and now it reads “TB patients’ stay” on page 5, line 93.*

**Comment:** In Line 28, please reword to “TB patient’s length of hospital stay and management, healthcare workers’ exposures to”.

***Response:*** *We have corrected this sentence accordingly. The revised sentence is, “This study aimed to assess PTB patients’ length of hospital stay and management and healthcare workers’ exposures to PTB patients, and the use of PPE in two public tertiary care hospitals in Bangladesh” on page 5, lines 95-97.*

**Methods:**
**Comment:** In Line 36 (near the top of Page 6), please change “experiences” to “experience”.

***Response:*** *We have corrected this on page 6, line 109.*

**Comment:** In Line 41, please replace "diagnostic" with "diagnosis".

***Response:*** *We have corrected this on page 6, line 115.*

**Comment:** In Line 42, at the end of the sentence, consider adding “within these facilities”, so that it reads “(there was already an established relationship and research occurring within these facilities)”.

***Response:*** *We have corrected this. Now it reads,* *“there was already an established relationship and research occurring within these facilities” on page 6, lines 115-116.*

**Comment:** In Line 44 (in the Observations section), how many adult general medicine wards did the team conduct observations in? I suggest adding in brackets “(n male wards and n female wards)”.

***Response:*** *The number of adult medicine wards and adult female medicine wards have been added. Now it reads, “After seeking written approval from the hospital directors and unit heads, the team conducted observation in adult general medicine wards (two male medicine wards and two female medicine wards)” on pages 6-7, lines 121-122.*

**Comment:** In line 57 (in the Hospital record review section), please remove “the” from “We reviewed the hospital records”.

***Response:*** *The word ‘the’ has been removed. Now it reads, “After seeking written approval from the hospital directors and unit heads, we reviewed hospital records from December 2017 to July 2019” on page 7, lines 137.*

**Comments:** In Line 59, please replace “registers” with “registries”. In Line 61, please replace “CXR” with “chest x-ray (CXR)”, as I believe this is the first time that the readers see this abbreviation.

***Response:*** *Thank you, we have revised the sentence. Now it reads, “Each day, the team members visited the adult medicine wards, reviewed the patient registries, and documented the number of presumptive TB patients on admission, number of sputa collected from suspected TB patients, number of suspected TB patients sent for a chest x-ray (CXR) (including the percentage with an abnormal CXR), …...” on page 7, lines 139-142.*

**Comment:** In Line 62, please replace “the” with “those” so that it reads “diagnosed with TB among those suspected”.

***Response:*** *Corrected accordingly on page 7, line 143.*

**Comment:** In Line 64, please add “testing” so that it reads “and the percentage testing positive”.

***Response:*** *Corrected accordingly. Now, it reads, “suspected TB patients sent to GeneXpert testing and the percentage testing positive” on page 8, line 145.*

**Comment:** In Line 65, please add “the” so that is reads “the team talked to the patient’s family”.

***Response:*** *We have corrected this on page 8, line 146.*

**Comment:** In Line 68 (in the In-depth interviews section), please replace “purposively” with “purposefully”

***Response:*** *The sentence has been revised. Now it reads, “Seventeen in-depth interviews were undertaken with purposefully selected senior and junior level HCWs from the medicine wards, labs and directly observed therapy, short-course (DOTS) clinics” on page 8, lines 149-151.*

**Comment:** In Line 76, please replace “them from tuberculosis” with “TB transmission”.

***Response:*** *Revised accordingly on page 8, line 159.*

**Comment:** In Line 81 (in the Analysis section), please replace “spend” with “spent”.

***Response:*** *Revised accordingly on page 8, line 166.*

**Results:**
**Comment:** In Line 100, please state whether the numbers pertain to male wards, female wards, or both.

***Response:*** *The sentence has been revised for clarity. The number pertains to per session per ward. Now it reads, “During the observation periods, there was a median of 86 (IQR: 164.75) occupants per session per ward” on page 10, lines 194-195.*

**Comment:** Please be consistent with bracket use – currently there are both square and circular brackets being used, i.e. [] and ().

***Response:*** *Thank you. The square brackets have been replaced with circular brackets throughout body of the manuscript.*

**Comment:** In Line 101, please add an IQR for the 10 HCWs.

***Response:*** *IQR added. Now it reads, “.. and 10 (IQR:10) HCWs” on page 10, line 196.*

**Comment:** In Lines 102-103, please consider rewording as follows “A median of 3.35 (IQR: 2.87) occupants were present per 10 m2 of floor space, with the highest number of occupants observed in female medicine wards in Hospital A; 7.0 (IQR: 0.7) occupants per 10 m2 of floor space”.

***Response:*** *The sentence has been revised. Now it reads,* *“A median of 3.35 (IQR: 2.87) occupants were present per 10 m^2^ of floor space, with the highest number of occupants observed in female medicine wards in Hospital A; 7.0 (IQR: 0.71) occupants per 10 m^2^ of floor space (Table 1)” on page 10, lines 197-200.*
**Comment:** In Line 106, please replace “was” and “and”, and change “them” to “whom”, so that it reads “The team recorded a median of 15 people (IQR: 15) coughing during the observation period, most of whom were patients”.

***Response:*** *Thank you. The sentence has been revised accordingly as* *“The team recorded a median of 15 people [IQR: 15] coughing during the observation period, most of whom were patients (median 8, IQR: 10) and their caregivers (median 6, IQR: 8)” on page 10, lines 202-204.*

**Comment:** In Line 109, please superscript the "2" in "10 m2".

***Response:*** *We have made suggested change.*

**Comment:** In Line 109, please add “of” so that it reads “Only 8.4% of”.

***Response:*** *Thank you. We have corrected this accordingly.*

**Comment:** In Line 118, please add “having” so that it reads “identified as having presumptive TB”.

***Response:*** *We have corrected this accordingly. Now it reads “identified as having presumptive TB” on page 11, line 215.*

**Comments:** In Line 118, please replace “and” with “, of which” so that it reads “over 20 months, of which 22%”. In Line 120, please add “were” so that it reads “55% were male”.

***Response:*** *We have corrected this accordingly. Now it reads, “There were 1,200 patients identified as having presumptive TB in the study wards over 20 months of which 22% (263/1200) were diagnosed with confirmed PTB. The median age of the patients was 52 (IQR: 30), and 44% (117/263) were male” on page 11, lines 215-217.*
**Comment:** In Lines 128-129, is there a reason why mean hospitalization duration was reported instead of median hospitalization duration?

***Response:*** *Thank you. Now, we used median hospitalization duration with IQR. Now it reads, “The median duration from hospital admission to final diagnosis was 4.6 days (IQR: 3.8 days), and to hospital discharge was 4.7 days (IQR: 3.8 days)” on page 12, lines 233-234.*

**Comment:** In Line 131, please add “that” so that it reads “Among 17 HCWs that participated”.

***Response:*** *Corrected accordingly on page 12, line 245.*

**Comments:** In Line 134, please reword as “The average age of the participants was 46.1 years”. Please also add a standard deviation to this. Is there a reason why mean was reported instead of median? Following Line 134, consider adding a sentence to this effect: “What follows is a thematic analysis of the in-depth interviews.”

***Response:*** *We have now used median with IQR. It reads, “The median age of the participants was 46 years (IQR:18), and the median years of experience in hospitals were 24 (IQR:17). The following is a thematic analysis of the findings from in-depth interviews” on page 12, lines 248-250.*

**Comment:** In Line 141, please change “as” to “with” so that it reads “six to eight patients with presumptive TB”.

***Response:*** *Revised accordingly. Now it reads, “Another participant highlighted that on admission day, they receive six to eight patients with presumptive TB” on page 13, lines 259-260.*

**Comment:** In Line 142, consider rewording as such: “Of these patients, only one or two are confirmed to have PTB disease”.

***Response:*** *Based on the suggestion, we have revised the sentence. Now it reads, “Of these patients, only one or two are confirmed to have PTB disease” on page 13, line 260.*

**Comment:** In Line 153 (in the TB patients diagnostic delay section), please replace “a” with “one” so that it reads “around one week”.

***Response:*** *We have corrected this. Now it reads, “The participants mentioned that it took around one week to diagnose a patient with TB” on page 13, line 271.*

**Comment:** In Lines 154-157, consider rewording as such: “Two of the participants (both doctors) mentioned that patients are often admitted in the ward after office hours (i.e. after 2:30 pm) and do not receive a prescription for a TB test until the doctors’ rounds on the following day. They added that several tests were required to confirm that a patient had TB disease.”

***Response:*** *Based on the suggestion, we have updated the sentences. Now, they read, “Two of the participants (both doctors) mentioned that patients are often admitted in the ward after office hours (i.e., after 2:30 pm) and do not receive a prescription for a TB test until the doctors’ rounds on the following day. They added that several tests were required to confirm that a patient had TB disease” on page 13, lines 271-275.*

**Discussion:**

**Comment:** In Line 187, consider rewording as such: “This study identified that public tertiary care general hospitals admit PTB patients on a regular basis”.

***Response:*** *Thank you. Now it reads, “This study identified that public tertiary care general hospitals admit PTB patients on a regular basis” on page 15, line 309.*

**Comment:** In Line 190, please replace “ward” with “wards”.

***Response:*** *We have revised it on page 15, line 313.*

**Comment:** In Line 191, please add “Further” and “which”, so that it reads “Further, there was no use of masks among presumptive B patients, which allowed”.

***Response:*** *We have corrected this. Not it reads, “Further, there was no use of masks among presumptive TB patients, which allowed the patients with pulmonary or laryngeal TB to contaminate the wards’ air” on page 15, lines 314-315.*

**Comment:** In Line 194, please replace “supporting” with “supported”.

***Response:*** *We have revised it on page 15, line 317.*

**Comment:** In Line 208, please replace “Besides” with “Additionally”.

***Response:*** *We have corrected it on page 16, line 342.*

**Comment:** Line 218 (“natural ventilation could be more effective than mechanical ventilation if not appropriately maintained”) is unclear. Do you mean that, if mechanical ventilation is not appropriately maintained, natural ventilation is more effective at maintaining air flow?

***Response:*** *The sentence has been corrected accordingly. Now it reads, “Although natural ventilation depends on outdoor weather conditions such as direction and magnitude of airflow, in some contexts, such as when mechanical ventilation systems are not appropriately maintained, it may be more effective at maintaining airflow” on page 17, lines 357-358.*

**Comment:** In Line 222, please reword to: “…could not be ensured due to the closed windows and doors”. In Line 223, please replace “close” with “closed”.

***Response:*** *We have corrected this accordingly. Now it reads, “The minimum of 6 to 12 air change per hour required to control airborne infection control in the ward could not be ensured due to the closed doors and windows. Even if the windows are closed on one side that prevents cross ventilation, large openings on the other side may not be inadequate to maintain the minimum air change” on page 17, lines 361-365.*

**Comment:** In Line 229, please add “and” so that it reads “chest X-ray and had to wait”.

***Response:*** *We have added “and”. Now it reads, “First, the patients who arrived in the hospital after working hours could not manage a prescription for sputum or CXR and had to wait for a prescription until the next day” on page 18, lines 368-370.*

**Comment:** In Line 235, please add “a” so that it reads “had a chest X-ray”. In Line 236, please replace “that delay” with “which delayed”.

***Response:*** *Corrected accordingly. Now it reads, “Finally, although most of the patients had a CXR suggestive of PTB, the HCWs often had to wait for sputum test results to finalize the TB diagnosis, which delayed the discharge or TB treatment” on page 18, lines 374-377.*

**Comment:** In Line 238, please add “and”, “which” and “ward” so that it reads: “…remained undiagnosed for nearly a week, and exhaled mycobacterium tuberculosis through coughing and sneezing, which contaminated the ward environment”. Consider adding a sentence after this that mentions that many patients did not cover their coughs or sneezes.

***Response:*** *We have revised the sentence. Now it reads, “Due to all these health systems barriers, the PTB patients remained undiagnosed for nearly a week, and exhaled mycobacterium tuberculosis through coughing and sneezing, which contaminated the ward environment as many patients did not cover their coughs and sneezes” on page 18, lines 377-382.*

**Comment:** In Line 244, please replace "clinic" with "clinics".

***Response:*** *Revised accordingly on page 19, line 395.*

**Comment:** In Line 245, please replace “indicative” with “indicator”.

***Response:*** *We have corrected this accordingly on page 19, line 396.*

**Comment:** In Line 250, please replace “cough” with “coughs”.

***Response:*** *We have corrected this accordingly on page 19, line 401.*

**Comment:** In Line 253, please replace "close" with "closed".

***Response:*** *We have corrected this accordingly. Now it reads, “The ward occupants’ long exposures to contaminated air space, possibly poor ventilation due to closed doors and windows, and lack of airborne IPC implementation in hospital wards all created an environment that increased the risk of TB transmission” on page 19, lines 403-405.*

**Comment:** In Line 261, please add “an” so that it reads “If an isolation room”. In Line 263, please add “a” so that it reads “or a veranda”.

***Response:*** *We have corrected this accordingly. Now it looks, “If an isolation room is not possible, hospitals should separate presumptive TB patients either in the corner of the ward near windows with adequate natural ventilation or a veranda with an open window to the outside” on page 20, lines 412-414.*

**Comment:** In Line 268, please replace “similar intervention had” with “similar interventions have”.

***Response:*** *We have corrected this accordingly. Now it reads, “Similar interventions have been successful in identifying presumptive TB patients quickly in general hospitals” on page 20, lines 418-420.*

**General Comments**

**Comment:** On the title page, you have written “Key-wards” instead of “Key Words”.

***Response:*** *We have corrected this as “Keywords” on page 1, line 17.*

**Comment:** Please be consistent with your use of “inpatient” or “inpatient”.

***Response:*** *We have replaced “in-patient” with “inpatient” throughout the manuscript.*

**Comment:** Please be consistent with number formatting. In general, if the number is less than 10, you can write it out, e.g. “two” or “three”. If it’s at the beginning of a sentence, it can be written out as well, even if it’s larger than 10. In all other instances, it can be referred to numeric format, e.g. “15” or “25”.

***Response:*** *We checked the manuscript and corrected it. However, the median numbers and the IQRs that are less than 10 have not been spelled out.*

**Comment:** Please capitalize "iccdr,b".

***Response:*** *Thank you. icddr,b does not use capital letters for its acronym’ icddr,b’.*

**Comment:** Since you’ve already specified early in the paper that chest x-ray is abbreviated as “CXR”, any time it is mentioned afterwards, it can be referred to as CXR instead of chest x-ray.

***Response:*** *We replaced all “chest x-ray” with “CXR” after defining it once.*

**Comment:** When referring to the hospitals, please use “Hospital A” and “Hospital B” instead of “hospital A” and “hospital B”.

***Response:*** *Corrected accordingly throughout the manuscript.*

**Tables and Figures:**

**Comment:** Please add standard deviation to average number of HCWs per ward in Table 1.

***Response:*** *Now median number with IQR has been presented in Table 1 for all variables.*

**Comment:** Do the numbers in square brackets in the “Average number of windows closed” row represent standard deviation? If so, please add a footnote to this effect in Table 1.

***Response:*** *Now median number with IQR has been presented in Table 1.*

**Comment:** The x-axis of Figure 1 should read “Observation dates” instead of “Observations dates”.

***Response:*** *We now used “Observation dates” in figure 1.*

 **Major Comments**

**Comment:** The three-pronged approach to answering your research question is interesting, holistic, and engaging to the reader. However, a reader that is not familiar with mixed methods may not understand the rationale behind doing observations, chart reviews, and in-depth interviews. I suggest adding a brief section – it could be separate or integrated with your “Study design, study site, and data collection” section – that explains why each of the three methods was chosen, and what specific objectives each of the three methods would meet.

***Response:*** *As suggested by the reviewers, we have added following paragraph. “The use of these multi-method data collection tools allowed us to triangulate and cross-check the data that emerged from various sources[1]. This mixed-method approach also allowed to compare quantitative and qualitative data that increased the rigor of the research, which was less likely to be achieved using a single data collection tool[2]” on page 6, lines 105-108.*

Under observation section, we added following statement, “*The aim of observation was to systematically document patients and HCWs’ behaviors and practices occurring in a naturalistic environment[3]” on page 7, line 122-123.*

*Under in-depth interview section, we added- “Four field team members with several years of experience in qualitative research conducted the interviews to gain an understanding of the existing PTB management practices along with what precautions they took to prevent TB transmission in the hospitals” on page 8, line 152-153.*

**Comment:** The authors do a great job of summarizing their quantitative data using descriptive statistics. However, there are some questions that remain unanswered, which would help strengthen their conclusions. For example, are there important differences between Hospital A and B that limit their comparability? Are there important differences between characteristics of male and female wards? Are there factors that are statistically associated with a higher number of TB patients per ward? Are there factors statistically associated with delayed diagnosis? These are just examples of questions that would help strengthen the authors’ recommendations at the end if answered. I suggest adding some additional analysis to accompany the quantitative results (e.g. regression).

***Response:*** *Thank you for comments. We would like to clarify that the study objective was not to assess factors statistically associated with PTB patients’ length of hospital stay, and therefore, we have very few variables to evaluate. Based on reviewer’s suggestion, we did the additional analysis and updated the methods, results, and discussion section.*

*Under methods, we added, “We used logistic regression to estimate odds ratios with 95% confidence intervals (CIs) to determine factors associated with the higher length of hospital stay. The PTB patients’ median length of hospital stay was 4.7 days. To form a dichotomous outcome, patients who stayed 4.7 days or less in the hospital was coded as ‘0’ and patients who stayed more than 4.7 days was coded as ‘1’ and compared. Factors with a p-value <0.20 in the binary logistic regression analyses were included in a multiple logistic regression model to adjust for confounders” on page 9, lines 171-176.*

*Under the result section, we added, “**In the univariate analysis, PTB patients’* *“length of hospital stay” significantly differed by hospitals, age, and history of co-morbidities. In the multiple regression analysis, patients in Hospital A were two and a half times more likely to stay in the hospital greater than the median 4.7 days than patients in Hospital B (aOR -2.67, 95% CI:1.46-5.06). Patients aged 50-60 were also statistically significantly associated with a higher length of hospital stay (aOR3.06, 95% CI: 1.37-7.12). Moreover, patients with co-morbidities were 4.46 times more likely to stay greater than the median 4.7 days when compared with patients who did not report any comorbidity (aOR4.46, 95% CI:1.37-17.85) (Table 2)” on page 12, lines 235-242.*

*In the discussion section, we added, “The pulmonary TB patients’ length of hospital stays significantly differed by hospitals which could be due to their reliance on different TB diagnostic methods. The findings showed that Hospital A mostly relied on CXR and sputum smear results for diagnosis, whereas Hospital B mostly relied on GeneXpert and CXR results, which minimized the diagnostic delay. As shown in figure 2, GeneXpert thus facilitated reducing PTB patients’ length of hospital stay, and this finding concurs with prior published literature[4]. The patients in the age group 50 to 60 stayed longer in the hospital when compared with the age group 30 and below, and this could be because older patients with PTB often do not present the typical signs and symptoms that delay diagnosis and increase the length of hospital stay[5]. PTB patient with co-morbidities is a known risk factor for increased length of hospital stay, and our findings further confirm this hypothesis[6]” on pages 18-19, lines 383-392.*

**Comment:** In the discussion, the authors talk about generalizing conclusions to the broader healthcare context in Bangladesh and potentially other resource-limited settings. How representative are Hospital A and Hospital B compared to other tertiary hospitals in Bangladesh? I suggest adding a sentence or two to this effect. A good place to put this information would be the end of your Methods section’s first paragraph (where the authors provide rationale for selecting these hospitals).

***Response:*** *Now we added more information to the paragraph- “Moreover, these two hospitals were the largest tertiary care public hospitals that serve around 27 million people from 14 districts in Rajshahi and Barishal divisions in Bangladesh[7-10]” on page 6, lines 116-118.*

**Comment:** At (or near) the beginning of the discussion, I suggest briefly listing the main takeaway messages from each of the three approaches (observations, hospital record review and in-depth interviews). This will help the reader tie everything together before you proceed to discuss further detail.

***Response:*** *Thank you. Now, we added, “**The high density observed in the inpatient wards along with a higher number of presumptive TB patients could facilitate the transmission of mycobacterium tuberculosis though uncovered coughing, sneezing and talking in the hospital [11]. A very limited use of respiratory protection among HCWs and patients was also observed, increasing the risk of TB exposures among ward occupants in the hospitals. The hospital record reviews revealed a higher number of PTB patients in Hospital B. Patients in Hospital B, patients aged between 50-60 years and patients with co-morbidities were statistically significantly associated with higher duration of hospital stay (>4.6 days). The findings from indepth interviews revealed the context for admitting PTB patients in these hospitals along with challenges these hospitals pose to follow recommended TB IPC measures. Thus, public tertiary care hospitals may facilitate the transmission of TB among HCWs and other wards occupants and necessitate the immediate implementation of TB IPC measures” on page 16, lines 322-332.*

**Comment:** In the “Hospital record review” section of the results, you refer to Figure 2, which is a great depiction of timelines. The authors show in the picture that there are some areas for potential intervention, but don’t really touch on this in the text. I suggest adding a sentence or two to mention what kinds of interventions could be implemented at different time points, with reference to Figure 2.

***Response:*** *We now added, “**Our study also found that smear-based diagnosis increased the diagnostic delay as it required multiple samples. We thus recommend testing all presumptive TB patients with GeneXpert. We acknowledge that GeneXpert is expensive (USD 50 per test); however, the use of GeneXpert will reduce the length of hospital stay from several days to hours and, thus, will be more cost-effective. We also recommend a patient with PTB should be discharged within an hour from final diagnosis, or PTB patients should be isolated immediately after diagnosis confirmation” on page 20, lines 421-427.*

**Reviewer #2:**

**Comments:** The study appears to be sound, a statistical description of the quality of care for TB patients, reporting a structured observation that identifies the critical points of intervention, such as reinforcing education for the proper use of protective equipment, both for the health personnel as well as on the part of the patients, and the little application of hygienic measures by the patients. The results show the obvious need to monitor hospital overcrowding and the proper operation of naturally ventilated wards.

***Response****: Thank you for your valuable comments and appreciation.*

**Comments:** The statistical analysis and process is adequate, however, it is necessary to review the figures and their relevance in the conclusions. For example, figure 1 is only cited once in the results and does not impact the discussion.

***Response:*** *Based on your suggestion, we now interpreted the figure 1 findings in the discussion section. We added, “**Moreover, the number of family caregivers was twice the number of patients, making the inpatient ward more crowded. Low or no use of facemasks among family caregivers’ not only put them at risk of Mycobacterium tuberculosis exposures but also increases the possibilities of transferring the TB bacteria or other viruses to the ward occupants[12]” on 17, lines 344-347.*

**Comments:** In addition, the information is not clear, this figure indicates on the x-axis that the observations were in a period of two months from December 15 to 15 February, but the study lasted 2 years (December 2017 to July 2019). I recommend reviewing this aspect and possibly considering substituting for the thematic map used in the study.

***Response:*** *We would like to clarify that this study had multiple components: the hospital record reviews lasted two years. However, the observation was conducted for two months. The data presented in figure 1 was from the observation data, and therefore, the observation months has been shown on X-axis in figure 1.*

Bibliography

1.	Williams CM: **The identification of family members' contribution to patients' care in the intensive care unit: a naturalistic inquiry**. *Nursing in critical care* 2005, **10**(1):6-14.

2.	Wisdom J, Creswell oW: **Mixed Methods: Integrating Quantitative and Qualitative Data Collection and Analysis While Studying Patient-Centered Medical Home Models**. In*.*, vol. AHRQ Publication No. 13-0028-EF. Rockville, MD: Agency for Healthcare Research and Quality; 2013.

3.	Mays N, Pope C: **Qualitative research: Observational methods in health care settings**. *BMJ* 1995, **311**(6998):182-184.

4.	Chaisson LH, Duong D, Cattamanchi A, Roemer M, Handley MA, Schillinger D, Sur M, Pham P, Lin MA, Goldman LE *et al*: **Association of Rapid Molecular Testing With Duration of Respiratory Isolation for Patients With Possible Tuberculosis in a US Hospital**. *JAMA Intern Med* 2018, **178**(10):1380-1388.

5.	Thomas TY, Rajagopalan S: **Tuberculosis and Aging: A Global Health Problem**. *Clinical Infectious Diseases* 2001, **33**(7):1034-1039.

6.	Baty F, Schoch O, Tonko S, Brutsche M: **Comorbidity clusters associated with length of hospital stay in patients with tuberculosis**. *European Respiratory Journal* 2019, **54**(suppl 63):OA2133.

7.	Wikipedia: **Rajshahi Division**. In: *In Wikipedia.* vol. 2021. <https://en.wikipedia.org/wiki/Rajshahi_Division>: Wikipedia; 2021.

8.	Wikipedia: **Barisal Division**. In: *In Wikipedia.* vol. 2021. <https://en.wikipedia.org/wiki/Barisal_Division#Administration>: Wikipedia; 2021.

9.	Begum F, Alam S, Hossain A: **Funds for treatment of hospitalized patients: evidence from Bangladesh**. *Journal of health, population, and nutrition* 2014, **32**(3):465-470.

10.	Ferdos J, Rahman MM: **Maternal experience of intimate partner violence and low birth weight of children: A hospital-based study in Bangladesh**. *PloS one* 2017, **12**(10):e0187138.

11.	Turner RD, Bothamley GH: **Cough and the transmission of tuberculosis**. *The Journal of infectious diseases* 2015, **211**(9):1367-1372.

12.	Barker J, Stevens D, Bloomfield SF: **Spread and prevention of some common viral infections in community facilities and domestic homes**. *Journal of applied microbiology* 2001, **91**(1):7-21.
